# Evaluation of an Inexpensive Growth Medium for Direct Detection of *Escherichia coli* in Temperate and Sub-Tropical Waters

**DOI:** 10.1371/journal.pone.0140997

**Published:** 2015-10-23

**Authors:** Robert E. S. Bain, Claire Woodall, John Elliott, Benjamin F. Arnold, Rosalind Tung, Robert Morley, Martella du Preez, Jamie K. Bartram, Anthony P. Davis, Stephen W. Gundry, Stephen Pedley

**Affiliations:** 1 UNICEF, New York, United States of America; 2 School of Chemistry, University of Bristol, Bristol, United Kingdom; 3 Department of Civil and Environmental Engineering, University of Surrey, Guildford, United Kingdom; 4 Division of Epidemiology, School of Public Health, University of California, Berkeley, United States of America; 5 CPI International, Santa Rosa, CA, United States of America; 6 Public Health England, Bristol, United Kingdom; 7 Council for Scientific and Industrial Research, Pretoria, South Africa; 8 The Water Institute, University of North Carolina, Chapel Hill, NC, United States of America; 9 Brightwater Diagnostics Limited, Bath, United Kingdom; Catalan Institute for Water Research (ICRA), SPAIN

## Abstract

The cost and complexity of traditional methods for the detection of faecal indicator bacteria, including *E*. *coli*, hinder widespread monitoring of drinking water quality, especially in low-income countries and outside controlled laboratory settings. In these settings the problem is exacerbated by the lack of inexpensive media for the detection of *E*. *coli* in drinking water. We developed a new low-cost growth medium, aquatest (AT), and validated its use for the direct detection of *E*. *coli* in temperate and sub-tropical drinking waters using IDEXX Quanti-Tray^®^. AT was compared with IDEXX Colilert-18^®^ and either EC-MUG or MLSB for detecting low levels of *E*. *coli* from water samples from temperate (n = 140; Bristol, UK) and subtropical regions (n = 50, Pretoria/Tshwane, South Africa). Confirmatory testing (n = 418 and 588, respectively) and the comparison of quantitative results were used to assess performance. Sensitivity of AT was higher than Colilert-18^®^ for water samples in the UK [98.0% vs. 86.9%; p<0.0001] and South Africa [99.5% vs. 93.2%; p = 0.0030]. There was no significant difference in specificity, which was high for both media (>95% in both settings). Quantitative results were comparable and within expected limits. AT is reliable and accurate for the detection of *E*. *coli* in temperate and subtropical drinking water. The composition of the new medium is reported herein and can be used freely.

## Introduction

The WHO/UNICEF Joint Monitoring Programme for Water Supply and Sanitation estimates that 663 million people do not have access to an improved source of drinking water [1]. A far greater number, 1.8 billion [2], are thought to be exposed to faecal contamination and drink water that fails to meet health guidelines [3]. Ensuring the safety of drinking water is an enormous challenge and is not aided by the difficulties in assessing water quality. This is especially the case for microbial water quality, the principal drinking water contaminant of public health concern in most countries [4].

Assessing the microbial quality of drinking water by means of bacterial cell culture is well established and has been applied since the early 1900s [5]. The major technological changes have been the introduction of quantification by multiple tube fermentation, development of membrane filtration and more recently enzyme substrates [6]. Although many approaches can be used to detect faecal contamination, the majority are not in widespread use due to lack of regulatory approvals and the expense and complexity of many of the advanced approaches. Enzyme substrate tests offer a potentially simple approach even in remote areas of low-income countries [7], but most of the commercial kits are expensive ranging from $2 to $5 per test [8] and represent a substantial proportion of the overall costs of monitoring [9].

The market leaders in enzyme substrate tests are Colilert-18^**®**^ (C-18) and Colilert^**®**^ both manufactured by IDEXX. These media are included in the Standard Methods for the Examination of Water and Wastewater and UK “Blue book” along with more traditional methods [10,11]. Due to the expense of patent protected tests, traditional methods dominate the market, especially in many low-income and middle-income countries. These methods typically have two stages: the first provides a provisional result (taking up to 48 hours) that is confirmed by further tests that can take a further 20 to 24 hours. The confirmatory tests put pressure on incubation space and analyst time; and may thereby restrict the number of analyses that can be conducted in a given laboratory. Direct detection using enzyme substrates offers advantages in terms of its time to result and simplicity [7]. Moreover, enzyme substrates afford high specificity for *E*. *coli*, WHO’s recommended faecal indicator [3] and are considered the best available faecal indicator [12].

Given the high costs and logistical challenges of *E*. *coli* detection, researchers have developed several alternative tests. In resource limited areas the hydrogen sulphide (H_2_S) test [13] has become popular. Between 2004 and 2008 the government of India purchased over 8 million H_2_S tests for use in their monitoring programmes [14]. However, the test has its limitations, for example resulting in false positives in groundwater, and is not comparable with methods that detect and quantify *E*. *coli* [15], even when also used in a 100 ml test format [16]. Another alternative is the detection of thermotolerant coliform (TTC), often referred to as “faecal coliform”. TTC have advantages in terms of the simplicity and cost of the medium but control of the higher temperature incubation (44±0.5°C (ISO 9308.1:2000)) is critical to specificity and the test may not be reliable in settings where electricity is unavailable or intermittent. The development and ongoing use of these tests serves as a further demonstration of the constraints on monitoring and the demand for simpler and more affordable tests.

In evaluating the performance of media including for low- and middle-income countries, there is a need to assess representative water samples from the appropriate climate [17]. Although C-18 and other reference media have been found to perform well in temperate waters [18,19], they do not always perform as well in tropical and sub-tropical waters [20,21], presumably in part due to the predominant strains and water matrices which may not have been present in regions where the media were originally developed.

We set out to develop a simpler and lower cost test for *E*. *coli*. As part of this project, a novel growth medium aquatest (AT) was developed to address the limitations of other media and to reduce costs to a minimum without sacrificing performance. The medium has been designed to detect a single *E*. *coli* bacterium in a 100 mL drinking water sample and detection is achieved by fluorescence under ultraviolent (UV) light. The objective of this study was to evaluate the performance of AT in a variety of settings and using various strains of *E*. *coli*. AT was compared to C-18 and other reference methods in its ability to detect *E*. *coli* in temperate and sub-tropical environments. We report the composition of AT to encourage its use and increase access to inexpensive methods for the detection of *E*. *coli*.

## Methods

The methods used to assess the performance of AT compared to C-18 are outlined in Table 1. Unless otherwise specified, all materials were obtained from Sigma Aldrich.

**Table 1:** Summary of study locations and methods.

Study	Location	Sample types	Methods	Number of samples	Organizations	Analysis
Temperate	Bristol, UK (January to April 2010)	Natural water samples collected from wells, surface and wastewaters and spiked to concentrations in the range 1–100 *E*. *coli* per 100 mL. Surface water samples were collected from a combination of rivers, ponds and one lake. Five secondary effluent samples served as wastewater samples. See S1 Table for further details.	Aquatest, Colilert-18^®^, MLSB, and EC-MUG. Selected wells confirmed using EC-MUG and API-20E	140 replicates from 14 sources (418/430 confirmed)	Public Health England and University of Bristol	Sensitivity/ Specificity, difference vs. mean and Spearman rank correlation
Sub-tropical	Pretoria, South Africa (March to April 2010)	Sites were chosen by NRE staff based on historical knowledge of *E*. *coli* counts and the confirmed presence of non-target organisms. These included water from rivers (13), dams (5), ponds/lakes (3) boreholes (2), and secondary sewage effluent (2). See S1 Table for further details.	Aquatest and Colilert-18^®^. Selected wells confirmed using EC-MUG, API-20E & MacConkey Agar.	50 replicates from 25 sources (588 confirmed)	University of Surrey, Natural Resources & the Environment (NRE) unit at the Council for Scientific and Industrial Research (CSIR).	Sensitivity/ Specificity, difference vs. mean and Spearman rank correlation

### Aquatest medium

The composition of AT is given in Table 2. The medium was based on M9 Minimal Salts [22] as the components are cheap and widely available. A rich buffered nutrient medium, AT contains: cefsulodin to suppress some strains of *Pseudomonas* [23,24]; sodium dodecyl sulphate (SDS) to inhibit gram positive bacteria which could cause false positives [25]; 4-methylumbelliferyl-ß-D-glucuronide (MUG) as the substrate for the *E*. *coli* enzyme ß-glucuronidase producing 4-methylumbelliferone that can be detected using an ultraviolet lamp [26]; sodium pyruvate to support the recovery of chlorine injured bacteria [27,28]; and, sodium thiosulfate to neutralize chlorine in treated waters [29]. Complex nutrients casamino acids and yeast extract are included to provide amino acids, vitamins and minerals.

**Table 2:** Composition of AT *E*. *coli* Detection Medium.

Component		Conc.	Cost per test^b^ ^,^ ^c^
Name	Formula	(g/L)	(US cents)
Yeast extract	-	2.0	3.1
Casamino acids	-	1.0	5.4
Sodium sulphate	Na_2_S_2_0_3_	0.5	0.9
Sodium chloride	NaCl	0.5	0.5
Sodium phosphate dibasic	Na_2_HPO_4_	3.0	5.6
Sodium pyruvate	C_3_H_3_O_3_Na	0.1	1.3
Potassium phosphate monobasic	KH_2_PO_4_	1.5	2.3
Ammonium chloride	NH_4_Cl	1.0	0.7
Ammonium sulphate	(NH_4_)_2_SO_4_	1.0	0.7
Magnesium sulphate	MgSO_4_	0.25	0.4
Calcium chloride	CaCl_2_	0.05	0.3
Sodium dodecyl sulphate	C_12_H_25_NaO_4_S	0.1	0.6
MUG^a^	C_16_H_16_O_9_	0.05	4.0
Cefsulodin	C_22_H_21_N_4_O_8_S_2_	0.006	3.6
	**Total**	**11.06**	**29.5**

^a^4-Methylumbelliferyl-β-D-glucuronide

^b^Approximate cost per 100 mL test based on catalogue prices from Sigma Aldrich with the exception of MUG and cefsulodin for which prices are from BIOSYNTH

^c^Prices in 2015 US$.

Costs reported in Table 2 (calculations in S2 Table) are based on materials only and may not reflect the full costs of commercial production. Given the potential for great variability in the cost components of commercial production—from blending and packaging through QA/QC, regulatory approval and economies of scale of production—we make no attempt to estimate commercial cost.

For these studies, we used powdered AT blended by Neogen Corporation (Lansing, USA). The dehydrated medium was aseptically dispensed into 100 mL sample containers for mixing with samples prior to analysis. 100 mL aliquots of the drinking water samples were mixed with the AT medium until it dissolved and then incubated in an IDEXX Quanti-Tray^®^ (QT) at 37°C for 20 ± 2 hours. After incubation a UV lamp was used to indirectly detect the presence of *E*. *coli* based on *β*-glucuronidase hydrolysis of MUG.

### Growth curves

An *E*. *coli* National Collection of Type Cultures (NCTC) 9001 nutrient broth culture (20 hours incubation at 37°C) was adjusted to a McFarland standard of 0.5 and inoculated into a growth medium (300 μL) in chambers of a 96-well plate (NUNC) (estimated ~100 colony forming units (cfu)). Growth media used in this study were; AT, C-18 and Colilert^®^ and Colitag^TM^ (CPI International). Growth curves were generated by measuring the OD at 620 nm every 10 minutes for 24 hours at 37°C using a FLUOstar Omega (BMG Labtech). Triplicate samples were set up using three separate cultures and the mean values from these data were plotted together with their standard error.

### Temperate study

Testing was performed at Public Health England’s Bristol Laboratory which is accredited to ISO 17025:2005 by the United Kingdom Accreditation Service (UKAS). The Bristol Food, Water and Environmental Microbiology Laboratory took responsibility for overseeing quality assurance and quality control for this study.

#### Samples

According to US EPA guidelines for evaluating new drinking water test methods, *E*. *coli* detection media are assessed using samples with very low contamination levels (1–5 *E*. *coli* per 100 mL)[30]. Assessment for the comparability of Most probable Number (MPN) methods, however, is more appropriate at higher contamination levels. For comprehensive evaluation of AT performance in IDEXX QT, spiked samples were tested at both low (1–5 MPN/100 mL) and high MPN values (6–100 MPN/100 mL). Preliminary range-finding experiments were conducted using C-18 to ensure that bacterial counts in the samples being tested were within the ranges required for the evaluation.

Standard procedures were followed for sample collection [10]. Samples were collected and immediately placed on ice for transport to the laboratory. Permission to collect secondary effluent samples was given by Wessex Water. No permissions were required for other locations and activities. No endangered or protected species were involved in the field work of this study. Analysis of all samples took place within 30 hours of collection according to guidance from the US EPA [30]. Sterile sample collection bottles containing sodium thiosulphate (IDEXX) were used for any sample anticipated to contain chlorine residual for all other media.

#### Reference methods and confirmations

The performance of AT was compared to three established procedures for the determination of *E*. *coli* in drinking water: C-18 (IDEXX), LTB/EC-MUG (Oxoid), MLSB (Oxoid) and MacConkey Agar (Oxoid) [10,11]. C-18 was used in combination with the QT system (IDEXX). EC-MUG was used in a five tube ten-fold dilution format (5 x 10 mL, 5 x 1 mL and 5 x 0.1 mL) and MLSB using membrane filtration according to standard methods [11]. Incubation was at 37°C for 18 to 20 hours for C-18.

The US EPA recommends testing media on the basis of confirmatory testing by an independent method [30]; EC MUG was used as the independent method in this study for the comparison between AT and C-18. In cases where there was disagreement, a second method was used. API-20E was selected for its integrated multi-test format that provides a high level of confidence in bacterial identifications. In <5% of cases, the culture of interest was not available for API testing, so not all cases of conflict were resolvable by the independent method. Whenever these circumstances arose, the EC-MUG result was assumed to be correct.

Control strains were *E*. *coli* (NCTC 9001), non-*E*. *coli* total coliform (*Klebsiella pneumoniae* NCTC 9633) and non-coliform (*Pseudomonas aeruginosa* NCTC 10662). Strains were maintained on Microbank^TM^ beads at -80°C (-20°C in South Africa) and cultured overnight in nutrient broth (Oxoid) at 37°C before use. Control blanks of sterile deionized water were used with the CI-18 and media blanks were used with EC-MUG and MacConkey agar.

### Sub-tropical study

The principles and practice of ISO 17025: 2005 were applied in South Africa at the laboratory at the NRE and supported by the University of Surrey’s microbiological analytical laboratory, accredited to ISO 17025:2005 by the United Kingdom Accreditation Service (UKAS). Appropriate methods, forms and procedures were adapted from those used in the UKAS accredited laboratory.

#### Samples

To assess the performance of AT and C-18 in sub-tropical regions, water samples were taken in and around Pretoria, South Africa. Twenty-five samples were collected including water from Rivers (13), Dams (5), Ponds/Lakes (3) Boreholes (2), and Secondary Sewage Effluent (2). The sample sites were chosen by the NRE based on prior knowledge of *E*. *coli* counts and the confirmed presence of non-target organisms. No specific permissions were required for these locations and activities. No endangered or protected species were involved in the fieldwork of this study. Treated drinking water was not tested as it was considered unlikely to contain *E*. *coli*.

#### Reference methods and confirmations

In the sub-tropical study, C-18 and AT were compared against one another. Confirmations were conducted as described above except due to resource limitations, four positive and four negative wells were confirmed with EC-MUG from each AT/QT tray and two positive and negative wells from each C-18/QT tray. A greater number of confirmatory samples were taken for AT since C-18 has previously been described [19]. In cases of disagreement between AT or C-18 and EC MUG, a MacConkey agar streak plate, prepared from each QT well, was examined and a colony of each type was used to inoculate an API-20E (Biomerieux, Basingstoke, UK) identification strip.

### Data analysis

Protocols were developed prior to each study that detailed the data management process and statistical analysis and defined the procedures, including confirmatory testing. Data were entered into a spreadsheet prior to analysis in statitstical analysis software R (version 3.1.3).

#### Sensitivity and specificity

The US EPA recommends analyzing sensitivity and specificity rates in the context of confirmatory analyses [30]. Sensitivity (SN) is the proportion of samples contaminated that are correctly identified by the method [tp / (tp+fn)]. Specificity (SP) is the proportion of uncontaminated samples that are correctly identified by the method [tn / (tn+fp)]. Prior to pooling data to calculate global tests for differences, it was confirmed that the relationships were homogeneous across water samples using the Breslow-Day (B-D) test for homogeneity [30]. The B-D test has the null hypothesis that the odds ratio between the two measures is the same across all samples; a *p*-value greater than 0.2 in the B-D test suggests that it is reasonable to pool estimates of sensitivity and specificity across different water samples. In comparing SN and SP, Fisher’s Exact test was chosen rather than the Chi-Squared test because not all of the four cells had an expected count of greater than five. Exact binomial confidence intervals for the quantities were determined. Fisher’s Exact test was also used to assess whether there was any difference in performance between the media between settings.

#### Comparison of quantitative results

Quantitative results were expressed as the number of *E*. *coli* per 100 mL (either the number of cfu or MPN). Three samples in South Africa were greater than the upper limit of detection of the test formats (>200.5 for QT and >100 for membrane filtration); censored data were handled by setting them to half the lower limit of detection [31] or to the upper limit of detection. Quantitative results were compared using two methods: difference versus mean and correlation. Difference versus mean plots were used to assess whether there was any systematic variation in relative recovery with level of contamination [32,33]. Significant differences between methods were assessed using the Bradley Blackwood F-test [34]. Correlation between microbial counts was assessed using both linear regression and Spearman’s rank correlation coefficient. In all quantitative analyses, replicates in the South African study were treated as independent pairs.

## Results

### Growth curves

Comparison of growth curves for the *E*. *coli* positive control strain demonstrated comparable growth rates in AT, C-18 and Colitag^TM^ (S1 Fig). AT was more similar, in terms of lag phase and maximum optical density to C-18 than the 24 hour Colilert^®^ test.

### Sensitivity and specificity

Sensitivity of AT was found to be significantly higher than C-18 in both temperate and sub-tropical environments (Table 3); whereas 31 false negatives occurred across both studies for C-18, only four were identified for AT. There was no significant difference between the specificity of C-18 and AT in either temperate or sub-tropical waters (Table 3). No significant difference between settings was found for either medium (p>0.10).

**Table 3:** Sensitivity and specificity of AT and C-18 for the detection of *E*. *coli* in temperate and sub-tropical waters.

Study	Medium	n^d^	Sensitivity^e^	Fisher’s exact test^c^	n^d^	Specificity^e^	Fisher’s Exact test^c^
Temperate	AT	150	0.980^a^ [0.943–0.996]	<0.001	268	0.996^a^ [0.979–1.000]	1.000
	C-18	183	0.869^a^ [0.811–0.914]		247	0.996 [0.979–1.000]	
Sub-tropical	AT	194	0.995^b^ [0.972–1.000]	0.0030	198	0.980^b^ [0.949–0.995]	0.2723
	C-18	103	0.932^b^ [0.865–0.972]		93	0.957^b^ [0.894–0.988]	

^a^ Based on confirmed results using EC-MUG and API 20E

^b^ Based on confirmed results using API 20E and MacConkey Agar

^c^ Two sided Fisher’s Exact test for differences between aquatest and C-18

^d^ Number of confirmations

^e^ Confidence intervals are exact Binomial.

When analysis was restricted to samples containing only low concentrations of *E*. *coli* (1 to 10 *E*. *coli* per 100 ml), performance by both methods was unaffected, with AT producing a 3.0% false-negative rate and C-18 demonstrating a 13.1% false-negative rate. In sub-tropical waters, AT demonstrated 99.5% [95% CI: 97.2–100] sensitivity and 98.0% [95% CI: 94.9–99.5] specificity for *E*. *coli*.

False positives were uncommon in both temperate and sub-tropical waters using both AT and C-18 (<5%). Predominant isolates from false positive wells in South Africa were identified using API-20E (S3 Table).

### Quantitative performance

**Fig 1:**
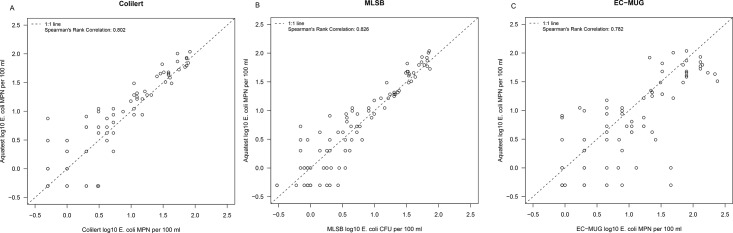
Scatter plots of aquatest *E*. *coli* estimates versus Colilert-18^®^, EC-MUG and MLSB in temperate water samples.

**Fig 2:**
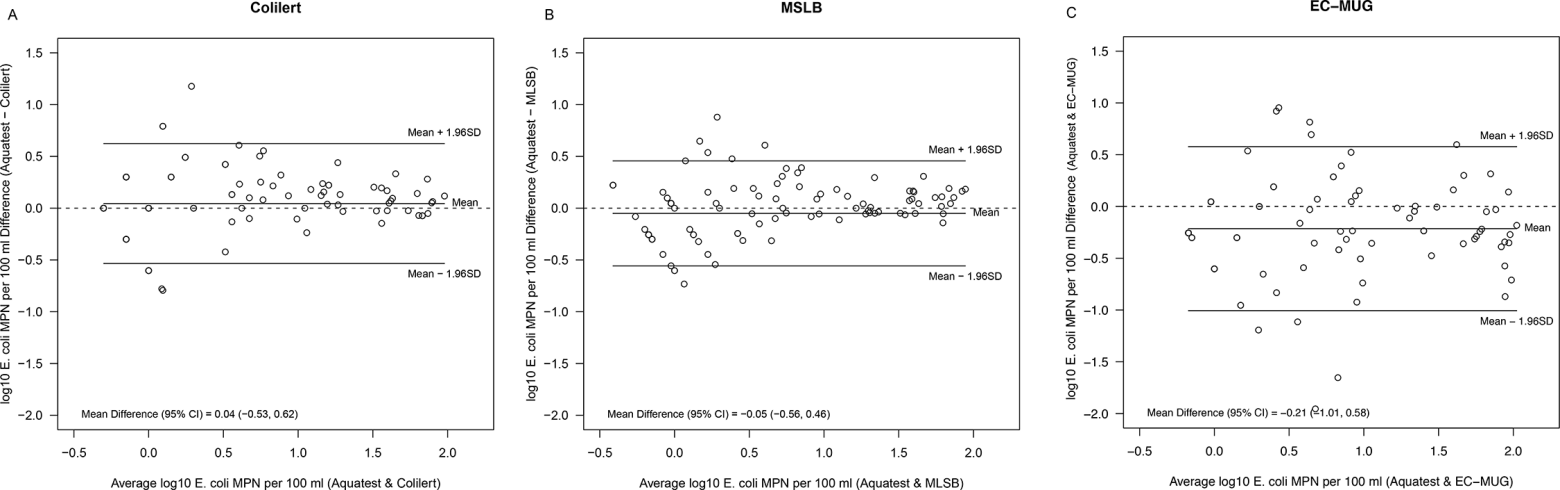
Difference versus mean plots for aquatest *E*. *coli* estimates versus Colilert-18^®^, EC-MUG and MLSB in temperate water samples.

**Fig 3:**
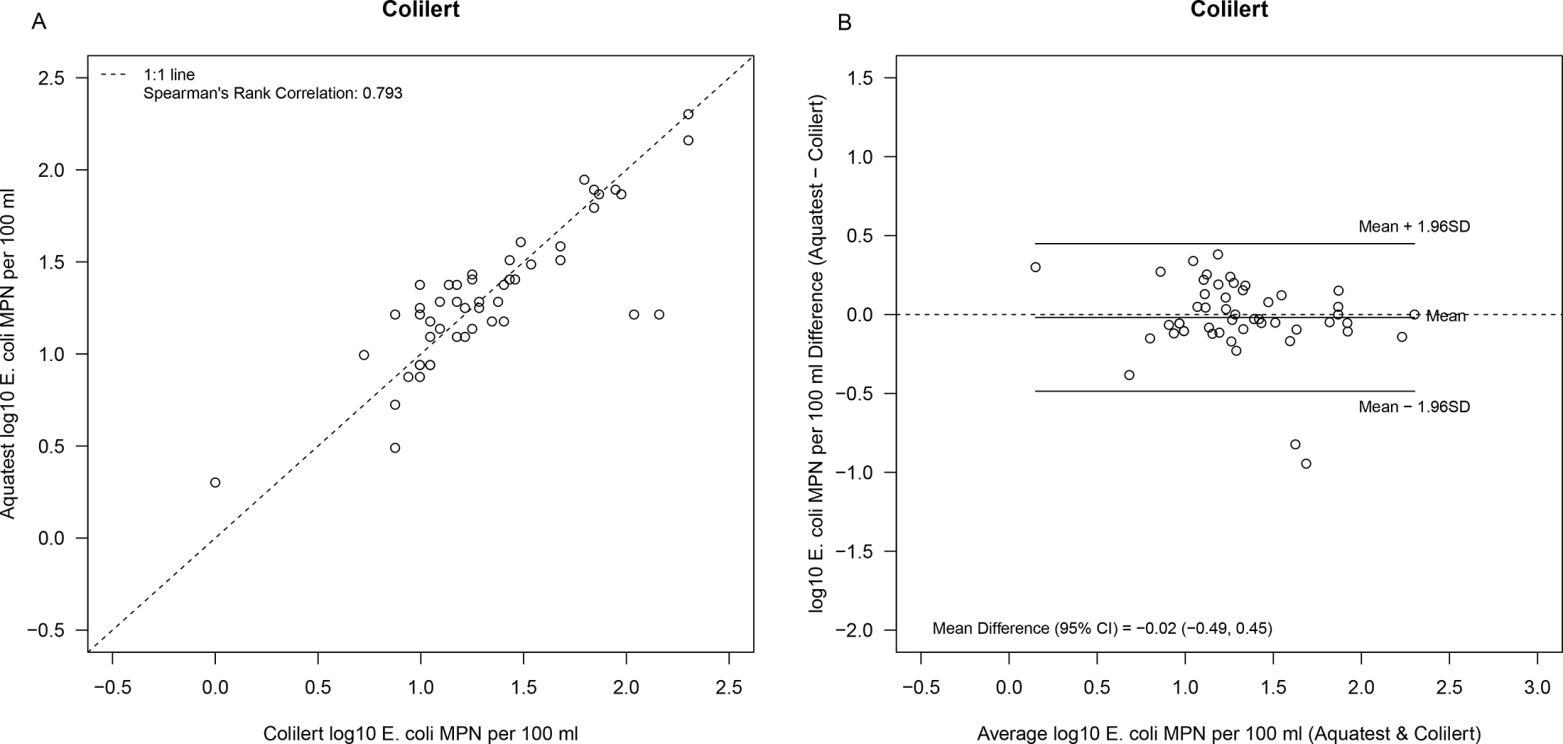
aquatest *E*. *coli* estimates versus Colilert-18^®^ (A) scatter plot and (B) difference versus mean in sub-tropical water samples.

Fig 1 shows scatter plots for AT compared to C-18, EC-MUG and MLSB. These show reasonable agreement between tests given the expected variability in counts. C-18, EC-MUG and the membrane filtration MLSB methods all demonstrated good correlation with AT (r^2^ = 0.802, 0.782 and 0.826 respectively). Fig 2 provides difference versus means graphs for the same comparisons. In contrast to results from the confirmatory analyses, no significant differences were observed in the levels of *E*. *coli* as measured using AT and C-18, EC-MUG or MLSB. In the sub-tropical study, the correlation between AT and C-18 was 0.88 (Fig 3A. Bradley Blackwood F-test (p = 0.55) found no difference between the media (Fig 3B).

## Discussion

### Development of AT

In developing AT we took into consideration issues such as physiological injury due to wastewater treatment, prolonged residence in natural waters and exposure to chlorine. We sought to develop an inexpensive medium that would perform comparably to reference methods including C-18 [35] and EC-MUG broth [24]. The formulation of AT was based on minimal medium M9 [36] which was chosen due to its simplicity and low cost. In this study we report the performance of AT used in IDEXX QT; however, it can also be used in presence/absence testing and alternative multiple tube formats such as the standard 3- or 5-tube ten-fold dilution series.

### Sensitivity and specificity

False negative results from a testing method can pose health risks when contamination in drinking water supplies is not detected. False positive results can also be problematic by eroding trust in the test method and by triggering unnecessary corrective actions that may waste resources. This is particularly problematic in settings where resources are severely stretched.

In both temperate and sub-tropical waters, AT was found to be sensitive and specific, producing very few false positives or false negatives. Sensitivity of AT in temperate waters was 98.0% [95% CI: 94.3–99.6] compared to 86.9% [95% CI: 81.1–91.4] for C-18. It can be stated with confidence that AT is at least as sensitive as one of the most widely known global market standards for indicator-based detection media, C-18.

Confirmatory analyses were based on EC MUG and API testing. All false negatives contained *E*. *coli* by definition. The organisms isolated from false positive wells in South Africa are summarized in S3 Table and in all cases at least one of the predominant isolates has been reported to have *β*-glucuronidase activity: *Citrobacter freundii* [37] and *Enterobacter cloacae* [38], *Klebsiella oxytoca* [39] and *Enterobacter sakazakii* [40]. In isolating predominant strains, it is possible that the bacteria responsible for false positives were not identified. Additional information may have been gained through re-testing these strains in C-18 and AT but that would not have ruled out the possibility of *E*. *coli* having originally been present in the sample but not recovered by the either medium.

Multiple studies have compared the performance of C-18 with alternative methods, but few of these have included samples from tropical or sub-tropical settings or low-income countries [17] despite the fact that C-18 is widely used in health surveillance, operational monitoring and research, including epidemiological trials. In temperate settings C-18 has often performed well in accurately detecting *E*. *coli* [18,19]. Studies in Taiwan, however, found C-18 to perform poorly and variably in sub-tropical freshwaters: Chao et al. [20] reported false positive and negative rates of 7.4% and 3.5% respectively, whereas a later study reported a substantially higher rate of false positives (36.4%) and higher false negatives (11%) [21]. In our study, we found sensitivity of C-18 to be slightly lower in the temperate setting (0.87) than the sub-tropical setting (0.93). The low sensitivity of C-18 relative to AT in temperate waters is consistent with previous reports that found Colilert^®^ to recover fewer pure strains of *E*. *coli* than other enzyme substrate methods [18,41].

### Quantification

When comparing overall detection rates amongst all the methods included in this study, correlation and difference versus mean analyses indicate that the methods show a high level of agreement. MLSB, EC-MUG and C-18 all demonstrated good correlation with AT (r^2^ = 0826, 0.782 and 0.802, respectively). Comparatively higher variability was seen at the lower concentrations of indicator bacteria, this is expected based on the larger variability associated with low spiking levels. Bacterial dispersion can affect detection rates if the concentration of the target microbe in a given sample is very low. The lowest correlation was seen for the comparisons between EC-MUG and other methods, suggesting that the test format may be more important than the medium in determining comparative performance. The EC MUG test format is 55.5 mL in total compared with 100 mL used in the other tests. With a smaller number of tubes covering a wider range than QT, EC MUG is expected to be less precise [42] and yield more biased MPN estimates [43]–as a result EC MUG counts are more variable and occasionally much higher than the other three methods.

### Limitations and future work

The test has been evaluated in two locations (Bristol, UK and Pretoria, South Africa) using a relatively small number of samples (<1000). We therefore recommend that the performance of AT be further compared with standard methods before adoption in a new setting or for alternative applications such as environmental monitoring. Further investigation of the performance of AT and other *E*. *coli* media could be enhanced by the use of a broader range of positive and negative controls, and polymerase chain reaction techniques—such information may enable further refinement of the medium.

As a freely available medium formulation, we recommend that regulatory agencies undertake the studies required to validate this medium for the detection of *E*. *coli*, as was done for EC MUG and MLSB. In some settings, regulatory requirements specify the use of total coliform testing in addition to *E*. *coli*. We have not included a total coliform indicator due to the additional cost and lesser sanitary significance compared to *E*. *coli* [12].

We were unable to identify an appropriate and cost-effective commercially available chromogenic substrate for *E*. *coli*. A variety of substrates are available [44] but these are generally expensive and large quantities are needed for a 100 ml test [45]. A promising option may be to use resorufin-β**-**D-glucuronide, which releases an intensely coloured dye and therefore can be used in smaller amounts [45]. A major potential advantage to the use of chromogenic substrates in an *E*. *coli* detection medium is that the antibiotic, cefsulodin, would not need to be included in the growth medium since this is required only to suppress bacteria such as *Pseudomonas fluorescens* which can interfere with visual detection of fluorescence [45]. An antibiotic-free medium would be less expensive to produce and would considerably increase robustness to temperature and moisture.

In this study, water samples were assessed up to 30 hours after collection following US EPA guidelines [30] whereas WHO guidelines recommend samples to be assessed within 24 hours or ideally 6 hours [3]. Previous studies have shown that recovery of bacteria can be affected by storage time [46,47] but this is likely to have little influence on relative performance of the different media. Furthermore, we did not assess the performance of AT in recovering chlorine-injured bacteria since two previous studies suggest it performs favourably compared with Colilert^®^ [45,48].

We evaluated the performance of AT during controlled incubation but the medium has also been evaluated at non-standard temperatures and shown to perform favourably when compared to Colilert^®^ [48]. Ambient temperature incubation [49], phase change incubation [48] and body incubation [50] all offer ways to reduce equipment requirements and facilitate the use of this medium in settings where electricity is not available or reliable [8]. Simple tests such as those based on flexible packing, for example the Compartment Bag Test [51], can provide basic quantification of *E*. *coli* levels. The use of AT in these and other field-deployable tests could enable more widespread water quality monitoring in resource-limited settings.

## Conclusions

We report the formulation of AT, an “open source” medium for the direct detection of *E*. *coli* in drinking water; it is hoped that this will enable its widespread use, refinement for different purposes and the development of new tests utilizing this inexpensive medium. AT is at least as sensitive as one of the most widely known global market standards for indicator-based detection media, C-18. AT showed consistently good performance in both a temperate and sub-tropical setting and has previously demonstrated ability to recover injured organisms. We therefore conclude that the medium is reliable and accurate for the detection of *E*. *coli*.

## Supporting Information

S1 Fig Growth of *E*. *coli* 9001 in Aquatest, Colilert^®^ and Colilert-18^®^ and Colitag^TM^ media. (PDF)

S1 Dataset aquatest, Colilert-18^®^, MLSB and EC-MUG results. (XLS)

S1 Table Sampling locations. (DOCX)

S2 Table Composition and cost of the aquatest *E*. *coli* Detection Medium. (XLSX)

S3 Table Organisms isolated from false positive and weak fluorescent wells in South Africa. (DOCX)
