# Supplementary material for: Evaluation of an Inexpensive Growth Medium for Direct Detection of Escherichia coli in Temperate and Sub-Tropical Waters
Source: PLoS One. 2015 Oct 23;10(10):e0140997. doi: 10.1371/journal.pone.0140997 (PMC4619692; doi:10.1371/journal.pone.0140997)

Figure S1. Growth of *E. coli* 9001 in aquatest, Colilert® and Colilert-18® media. Control strain *E. coli* 9001 (10^3) was inoculated into chambers of a 96-well plate that contained growth media, aquatest (●), Colilert® (♦), Colilert-18® (■) in triplicate. Cultures were incubated at 37°C for a total of 24 hrs, readings were taken at an optical density of 620 nm every 10 minutes. Mean values for each time point and standard error bars were plotted.

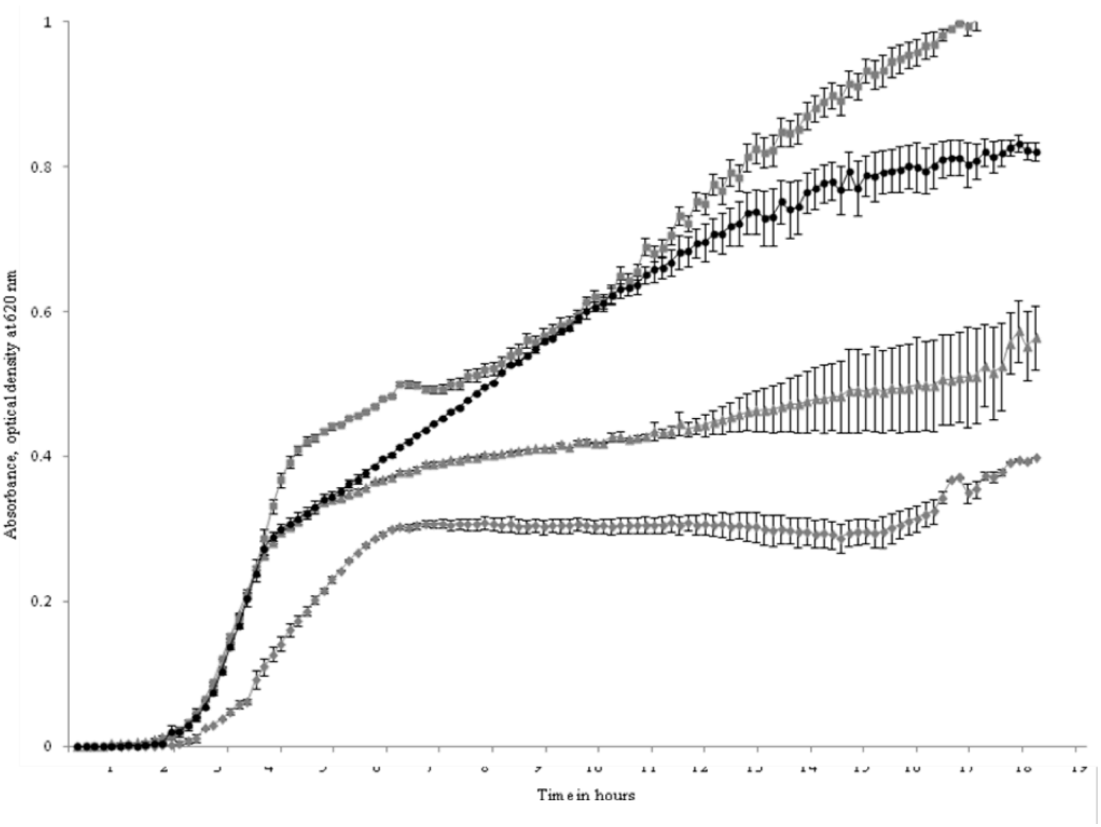

Supplement: S1 Fig — (PDF) [file pone.0140997.s001.pdf]
