# Supplementary material for: Evaluation of an Inexpensive Growth Medium for Direct Detection of Escherichia coli in Temperate and Sub-Tropical Waters
Source: PLoS One. 2015 Oct 23;10(10):e0140997. doi: 10.1371/journal.pone.0140997 (PMC4619692; doi:10.1371/journal.pone.0140997)
Supplement: S1 Table — (DOCX) [file pone.0140997.s003.docx]

**Supplementary information**

**Table S1. Sampling locations**

| **Sample** | **Location** | **Water type** |
| --- | --- | --- |
| *Temperate study, United Kingdom* | | |
| UK1 | Saltford, Somerset | Final effluent |
| UK2 | Keynsham, Bristol | Final effluent |
| UK3 | Thornbury, Bristol | Final effluent |
| UK4 | Kingston Seymour, North Somerset | Final effluent |
| UK5 | River Frome, Wade Street, Bristol | River |
| UK6 | Baltic Wharf, Bristol harbor | Surface water |
| UK7 | St Augustine’s reach, Bristol harbor | Surface water |
| UK8 | University of Bristol, Royal Ford garden | Pond |
| UK9 | St George’s Park Lake, Bristol | Lake |
| UK10 | Wood Farm, South Gloucester | Spring |
| UK11 | Hazel Brook, Blaise Castle, Bristol | Brook |
| *Sub-tropical study, South Africa* | | |
| SA1 | Zeekoegat Water Care Works | Sewage effluent |
| SA2 | Pienaars River, Kameeldrift | River |
| SA3 | Roodeplaat Dam | Dam |
| SA4 | Apies River | River |
| SA5 | Bon Accord Dam | Dam |
| SA6 | Daspoort Water Care Works | Sewage effluent |
| SA7 | CSIR Pond | Pond |
| SA8 | CSIR Northern Pond | Pond |
| SA9 | Moreletta Spruit, Garsfontein | River |
| SA10 | Rietvlei Dam | Dam |
| SA11 | Sesmyl Spruit | River |
| SA12 | Moreletta Nature Reserve | River |
| SA13 | Pienaars River | River |
| SA14 | Honde River | River |
| SA15 | Bronkhorst River | River |
| SA16 | Bronkhorstspruit | River |
| SA17 | Stinkwater, 1st House | Borehole |
| SA18 | Stinkwater, 2nd House | Borehole |
| SA19 | Soutpan spruit | River |
| SA20 | Struben Dam | Dam |
| SA21 | Irene River | River |
| SA22 | Centurion Lake | Lake |
| SA23 | Swartspruit | River |
| SA24 | Crocodile River | River |
| SA25 | Hartbeespoort Dam | Dam |
