# Supplementary material for: Evaluation of an Inexpensive Growth Medium for Direct Detection of Escherichia coli in Temperate and Sub-Tropical Waters
Source: PLoS One. 2015 Oct 23;10(10):e0140997. doi: 10.1371/journal.pone.0140997 (PMC4619692; doi:10.1371/journal.pone.0140997)
Supplement: S3 Table — (DOCX) [file pone.0140997.s005.docx]

**Supplementary information**

**Table S3: Organisms isolated from false positive and weak fluorescent wells in South Africa**

| Medium | Organisms |
| --- | --- |
| Aquatest | *Citrobacter freundii, Klebsiella pneumoniae* |
|  | *Enterobacter cloacae, Klebsiella pneumoniae* |
|  | *Citrobacter freundii* |
|  | *Enterobacter cloacae, Rahnella aquatilis* |
| Colilert^®^-18 | *Enterobacter cloacae, Enterobacter aerogens* |
|  | *Citrobacter youngae, Klebsiella oxytoca* |
|  | *Citrobacter koseri/farmer, Enterobacter sakazakii* |
|  | *Citrobacter freundii, Plesiomonas shigelloides* |
